# RNA N4-acetylcytidine modification in human cancers: from molecular function to oncogenic mechanisms

**DOI:** 10.1016/j.isci.2026.115080

**Published:** 2026-02-18

**Authors:** Yuchen Shi, Jiazhu Sun, Hong Chen, Kai Yu, Jiangfeng Li, Ben Liu

**Affiliations:** 1 Department of Urology, The First Affiliated Hospital, Zhejiang University School of Medicine, Hangzhou 310003, China

**Keywords:** Biochemistry, Epigenetics, Cancer

## Abstract

The RNA epitranscriptome represents a critical layer of gene regulation, with N4-acetylcytidine (ac4C) emerging as a pivotal modification in cancer biology. Catalyzed exclusively by N-acetyltransferase 10 (NAT10), ac4C decorates a broad spectrum of RNAs, profoundly influencing their stability and translation efficiency. This review synthesizes recent advances illuminating how the NAT10-ac4C axis drives oncogenic processes, including sustained proliferation, metabolic reprogramming, invasion and metastasis, immunosuppression, and therapy resistance by selectively stabilizing mRNAs encoding key oncoproteins. We detail the molecular mechanisms underpinning these roles across diverse malignancies, highlighting context-dependent functions and intricate cross-talk with other signal pathways. Furthermore, we explore the translational promise of this pathway, discussing NAT10 inhibitors and rational combination therapies that resensitize tumors to conventional treatments in preclinical models. Unraveling the full regulatory circuitry of ac4C will not only deepen our understanding of cancer pathogenesis but also pave the way for novel diagnostic and therapeutic strategies in precision oncology.

## Graphical abstract

**Figure:**
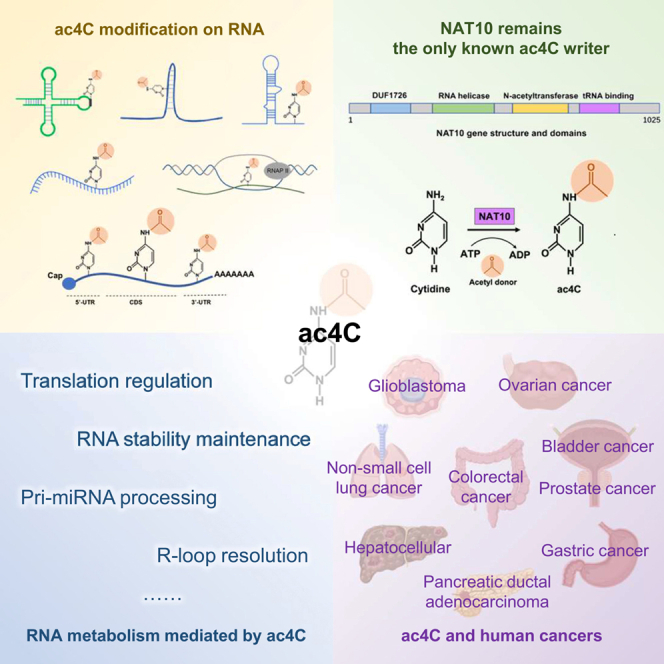

## Background

Recently, more attention has been paid to RNA chemical modifications in biological events for biologists. RNA metabolism mediated by post-transcription modification was defined as a novel field called “epitranscriptome.” Until now, the abundant RNA modification type N6-methyladenosine (m6A), N1-methyladenosine (m1A), N5-methylcytidine (m5C), and pseudouridine (Ψ) have been gradually identified to play a great role in RNA metabolisms.^1^ However, research on RNA acylation was rarely investigated until the finding of RNA N4-acetylcytidine (ac4C) in the regulation of RNA translation efficiency. RNA ac4C modification is a highly conserved RNA modification, which is the first acetylation event described among all RNA acylation types. Emerging evidence has proved that RNA modifications are comprehensively involved in the carcinogenesis and tumor progression of human cancers. In this review, we will conclude and show the novel insights into the potential role of RNA N4-acetylcytidine modification in human cancers.

## Molecular biology for N4-acetylcytidine

Since the 1980s, RNA ac4C modification has been detected to be conservatively distributed in tRNA and rRNA.^2^^,^^3^ With the development of detection techniques, ac4C modification in mRNA was also identified, and indicated a substantial regulatory role of mRNA stability and translation.^4^ Nowadays, N-acetyltransferase 10 (NAT10, also known as hALP) was the only identified ac4C writer. It has a long history of knowledge of NAT10 as an ac4C writer.

### N-acetyltransferase 10 from protein acetyltransferase to RNA acetyltransferase

Previously, NAT10 was first reported as protein acetyltransferase. NAT10, acting as a histone acetyltransferase-like protein, was involved in transcriptional activation and could enhance telomerase activity through the transactivation of hTERT promoter.^5^ Nucleus-localized NAT10 was also confirmed to maintain or enhance the stability of alpha-tubulin in the cell division process by acylating alpha-tubulin.^6^^,^^7^ In addition, NAT10 could acetylate P53 at K120 and stabilize P53 by counteracting Mdm2 to activate P53.^8^ PARP could be stabilized through the NAT10-mediated acetylation of PARP1 at lysine 949, which blocks its ubiquitination at the same residue.^9^ In addition, MORC2 is acetylated by NAT10 at lysine 767 (K767Ac), which regulates cell-cycle checkpoint control and resistance to DNA-damaging.^10^ More recently, NAT10 in hepatocellular carcinoma (HCC) promotes ACLY acetylation at K468, enhancing its stability and nuclear acetyl-CoA production, thereby activating the transcription of drug-resistance genes CYP2C9 and PIK3R1.^11^ In parallel, the NAT10-mediated acetylation of DDX21 at K236 and K573 enhances its helicase activity, promoting nucleolar R-loop resolution and genome stability.^12^ NAT10 undergoes liquid-liquid phase separation via its C-terminal domain, enabling the acetylation of the splicing factor SRSF2. This modification stabilizes SRSF2, which subsequently binds YTHDF1 pre-mRNA to induce exon 4 skipping and upregulate a truncated YTHDF1 isoform that drives gastric cancer cell proliferation and migration.^13^

Until 2014, Satoshi Ito et al. first demonstrated that ac4C is present at position 1773 in the 18 S rRNA of Saccharomyces cerevisiae. In addition, NAT10 acted as an RNA acetyltransferase to form ac4C 1773 site.^14^ In the same year, two acetylcytidines at positions 1297 and 1815 in the 3′ half of SSU rRNA mediated by NAT10 were also identified in fission yeast, Schizosaccharomyces pombe by comprehensive mass spectrometry-based analysis.^15^ It was also soon confirmed that in human NAT10, an ATP-dependent RNA acetyltransferase was responsible for the formation of ac4C at position 1842 in the terminal helix of mammalian 18S rRNA to involve in rRNA processing and ribosome biogenesis.^16^ Notably, the C/D box small nucleolar RNA SNORD13 is essential for the site-specific ac4C modification of 18S rRNA in both humans and zebrafish. However, genetic ablation of SNORD13 does not impair cell proliferation, pre-rRNA processing, or global protein synthesis in human cells, suggesting the existence of compensatory mechanisms or context-dependent functions of this modification.^17^ For tRNA, NAT10 was reported to specifically be responsible for the acetylation process with conserved adaptor THUMPD1.^18^ Transcriptome-wide mapping studies have elucidated that ac4C is selectively enriched within structural and non-coding RNAs (particularly rRNA and tRNA) and occurs predominantly at conserved 5′-CCG-3′ motifs.^19^

**Figure 1:**
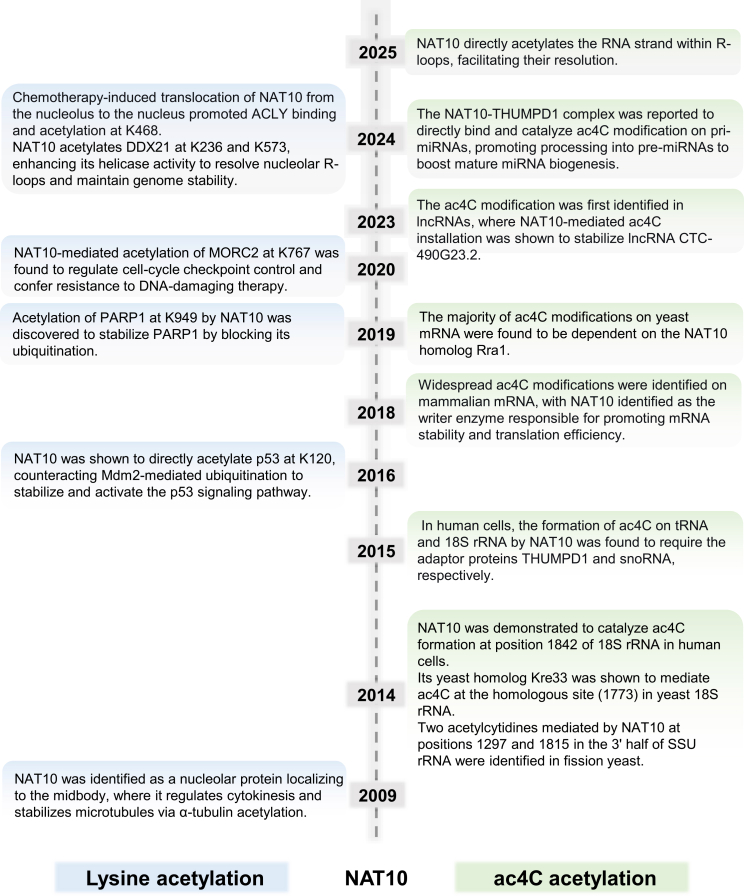
The functional evolution of NAT10: from protein acetyltransferase to RNA modification writer The schematic summarizes the expanding roles of NAT10, initially characterized as an acetyltransferase for protein substrates involved in transcriptional regulation, genome stability, and cell cycle control. Subsequent studies identified NAT10 as the canonical writer for ac4C across various RNA species, including 18S rRNA, tRNA, mRNA, and non-coding RNAs, where it regulates RNA stability, translation, and processing, thereby bridging protein and RNA acetylation in cellular physiology and disease.

In 2018, with RNA dot blot and liquid chromatography-tandem mass spectrometry assay, Daniel Arango et al. first described ac4C as an mRNA modification that is catalyzed by NAT10.^4^ Further transcriptome-wide mapping of ac4C revealed that discretely acetylated regions were enriched within coding sequences.^4^ Additionally, using acRIP-seq combined with BRIC-seq, they found that the stability and translation efficiency of ac4C-modified mRNA such as FUS and POLR2A, were significantly promoted by NAT10.^4^ Afterward, mRNA ac4C investigations were more focused. Guo et al. identified the unique ac4C-related transcripts, including USP18, GPX1, and RGL1, which regulate mRNA catabolic processes and translational initiation in immune and inflammatory responses for systemic lupus erythematosus disease.^20^ NAT10 was also found to promote osteogenic differentiation by mediating ac4C of Gremlin 1 mRNA degradation.^21^ More recently, researchers have found the presence of ac4C modifications on non-coding RNAs such as long non-coding RNAs,^22^^,^^23^ primary microRNAs.^24^ By reviewing the whole finding history of NAT10-mediated ac4C modification, we concluded that NAT10 is not only a protein acetyltransferase but also RNA acetyltransferase. This history of profound insight into NAT10 as ac4C writer was concluded as Figure 1.

### N-acetyltransferase 10 is responsible for RNA N4-acetylcytidine modification

**Figure 2:**
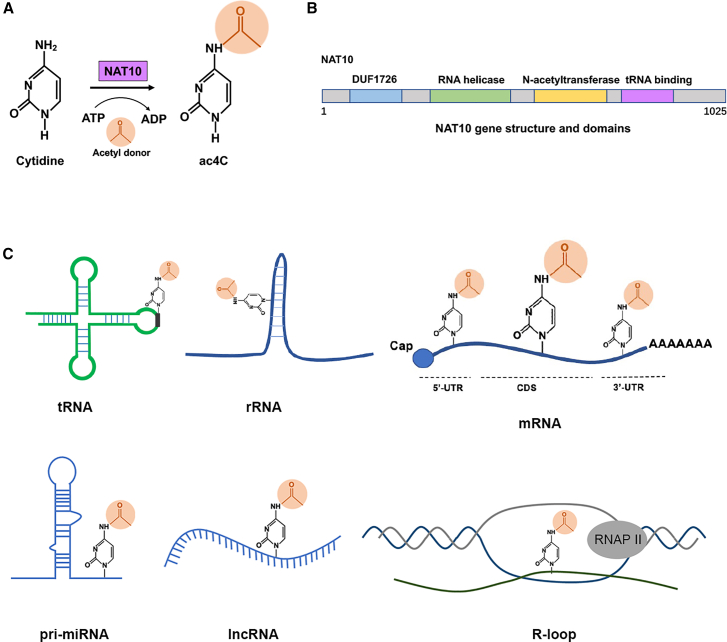
NAT10-mediated ac4C modification: catalytic mechanism, domain architecture, and RNA substrate diversity (A) The catalytic cycle of ac4C deposition by NAT10. NAT10 utilizes ATP and acetyl-CoA as cofactors to catalyze ac4C formation. This process is facilitated by specific adaptor proteins, including THUMPD1 for tRNA modification and small nucleolar RNAs for rRNA modification. (B) Domain architecture of human NAT10. The protein contains several functional domains: an N-terminal DUF1726 domain (function uncharacterized), a central helicase domain, an N-acetyltransferase domain responsible for catalytic activity, and a C-terminal RNA-binding domain. (C) The landscape of ac4C modification across RNA species. The schematic illustrates the presence of ac4C modifications on diverse RNA substrates, including tRNA, rRNA, mRNA, primary microRNAs, long non-coding RNAs, and R-loop structures, highlighting the broad regulatory scope of NAT10 in RNA biology.

So far, biologist found that NAT10 is the only RNA ac4C modification writer, using ATP and acetyl-CoA donor (Figure 2A), to interact with certain adaptors such as human THUMPD1 (for tRNA) or U13/snR4, SNORD13, snR45 snoRNAs (for rRNA) to together finish the ac4C catalytic process.^18^^,^^25^^,^^26^ In contrast to the well-characterized acetylation machinery, the identification of specific erasers for ac4C has been more elusive. Recent work has revealed that SIRT7, a nucleolar sirtuin, functions as an NAD^+^-independent deacetylase for ac4C on rRNA.^27^ However, the deacetylating activity of SIRT7 at the mRNA level requires further experimental validation, and its physiological significance and universality remain incompletely understood. Nowadays, for the NAT10 domain architecture, it has been found to be composed of the DUF1726 domain, RNA helicase domain, N-acetyltransferase domain, and RNA binding domain (Figure 2B).^4^^,^^28^ For basic details, we concluded a schematic graph of ac4C modification processing and NAT10 gene structure (Figure 2).

### N-acetyltransferase 10 is regulated by several factors

The expression or function of NAT10 is regulated by several factors, which all indirectly contribute to the regulation of ac4C-mediated RNA metabolism. All these regulations include processes of transcription, post-transcription, post-translational modification, and other unknown mechanisms.

The transcription regulation was important for NAT10 expression. The vital tumor driver oncogene c-MYC was found to directly activate NAT10 expression as a transcription factor to promote tumor progression in lung cancer.^29^ In hepatoblastoma, the transcriptional coactivator YAP1 binds directly to the NAT10 promoter, enhancing its expression.^30^ In the context of liver fibrosis and aging, SMAD2 and SMAD3 directly bind to the NAT10 promoter, enhancing its transcription and establishing a positive feedback loop in which NAT10, in turn, stabilizes TGFβ1 mRNA via ac4C modification.^31^

Among all regulatory factors, the self-modification of the NAT10 protein is the dominant regulation. The self-deacetylation of NAT10 protein mediated by SIRT1 was found to interfere with rRNA processing.^32^ Another finding also demonstrated the role of NAT10 protein self-acetylation in pre-rRNA transcription. It suggested that the lysine residue at 426 (K426) of NAT10 is crucial for the acetylation of upstream binding factor (UBF), which is responsible for recruiting PAF53 and RNA polymerase I to rDNA, eventually resulting in activating pre-rRNA transcription.^33^ DDX21 promotes NAT10 expression by competitively binding to the catalytic domain of SIRT7, which suppresses transcription by deacetylating histone H3K18 at the NAT10 promoter, thereby blocking H3K18 deacetylation and relieving transcriptional repression.^34^ Recently, lysine 2-hydroxyisobutyrylation of NAT10 (K823-Khib) mediated by KAT7 promoted the protein stability by recruiting USP39.^35^ Ubiquitination degradation of NAT10 mediated by E3 ubiquitin ligase, ZSWIM6 was inhibited by RNPS1, thereby promoting high NAT10 expression in head and neck squamous cell carcinoma.^36^ PARP1-catalyzed PARylation of NAT10 on three conserved lysine (K) residues (K1016, K1017, and K1020) within its C-terminal nucleolar localization signal motif (residues 983–1025) was responsible for its nucleoplasmic translocation after DNA damage.^37^ Additional post-translational modifications (PTMs) also regulate NAT10 activity, including lactylation. Lactylation of NAT10 at lysine 290 (K290) by α-tubulin acetyltransferase 1 (ATAT1) promotes ac4C modification on Kaposi’s sarcoma-associated herpesvirus (KSHV)-encoded tRNA^Ser−CGA-1-1^, thereby enhancing the translation of KSHV lytic transcripts and facilitating viral reactivation.^38^

Small RNAs, as important post-transcription regulators, were confirmed to be involved in NAT10 expression regulation. Liu et al. reported that miR-6716-5p could directly inhibit the NAT10 expression at both mRNA and protein levels by directly targeting 3′-UTR.^39^ A heart-apoptosis-associated piRNA (HAAPIR) was identified to directly interact with NAT10 and enhance the ac4C acetylation of downstream Tfec mRNA transcript to increase Tfec expression.^40^ In cervical cancer, the circular RNA circMAST1 directly binds to NAT10 and competitively sequesters NAT10, preventing its association with downstream mRNA and thereby reducing the ac4C-mediated stabilization of its transcripts.^41^

Translocation of NAT10 was reported by different factors. Zhang et al. demonstrated that the subcellular redistribution of NAT10 can be induced by decreasing in GSK-3beta activity, which increased colorectal cancer cell motility.^42^ Further mechanistic investigation found that the inhibition of GSK-3β promotes nuclear export of NAT10 in a CRM1 (nuclear export system) -dependent manner.^42^ Upon oxaliplatin treatment in HCC, NAT10 translocates from the nucleolus to the nucleoplasm, where it acetylates downstream protein targets to promote chemoresistance.^11^

**Figure 3:**
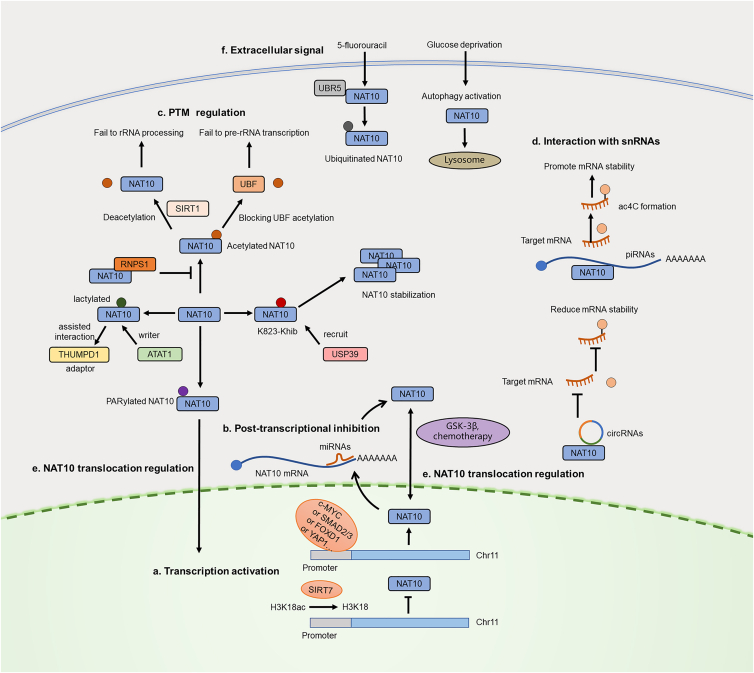
Multilayer regulatory network controlling NAT10 expression, activity, and subcellular localization This schematic illustrates the diverse molecular and signaling cues that regulate NAT10 across multiple layers, including transcriptional activation, post-transcriptional control, post-translational modifications, and subcellular translocation.

Extracellular cues and chemical agents significantly influence NAT10 expression and stability. Chemotherapeutic agents such as 5-fluorouracil promote the interaction between NAT10 and the E3 ubiquitin ligase UBR5, facilitating NAT10 ubiquitination and its subsequent degradation via the proteasome.^43^ Similarly, metabolic alterations regulate NAT10 stability, linking nutrient availability to epitranscriptomic control. In gastric cancer, glucose deprivation induces autophagy, promoting the interaction between NAT10 and the autophagic receptors SQSTM1 and LC3. This leads to NAT10 delivery to lysosomes for degradation, and a consequent reduction in ac4C modification.^44^ To sum up, all the above regulations were concluded in Figure 3.

## RNA metabolism mediated by N4-acetylcytidine

As with most RNA modifications, RNA metabolism was last to be induced for different biological functions or phenotypes. RNA ac4C modification was also responsible for various RNA metabolism events such as RNA stability, translation efficiency regulation, and so forth. Herein, we separately conclude each event in detail.

### Translation regulation

The first translation investigation was found in Escherichia coli. The wobble base of elongator tRNA(Met) is modified to ac4C, which was thought to ensure the precise recognition of the AUG codon by preventing the misreading of the near-cognate AUA codon.^45^

This foundational finding established ac4C as an evolutionarily conserved mechanism for safeguarding decoding fidelity, a principle that extends to eukaryotic systems. Cryo-electron microscopy was employed to resolve mammalian 80S initiation complexes assembled on mRNAs bearing site-specific ac4C at the −1 position within the Kozak sequence. This high-resolution structural analysis revealed that acetylation disrupts a key hydrogen bond between the 2′-OH of the cytidine ribose and the hypermodified t6A at nucleotide 37 of the initiator tRNA Met, thereby impairing start codon recognition and providing a mechanistic explanation for the repressive role of ac4C in canonical translation initiation.^46^

Beyond initiation, ac4C within coding sequences enhances translational elongation by stabilizing codon-anticodon interactions. The enrichment of ac4C at wobble positions in human mRNAs promotes efficient decoding, likely by optimizing base-pairing stability and reducing ribosomal stalling. This mechanism mirrors its ancestral role in tRNA and underscores ac4C as a versatile modulator of translation dynamics. Furthermore, site-specific acetylation may fine-tune local elongation rates, potentially influencing co-translational folding and chaperone recruitment, which is an emerging paradigm in epitranscriptomic regulation.^47^

### RNA stability maintenance

The increased effect of ac4C modification on mRNA stability has been mentioned above.^4^ While the initial characterization of ac4C highlighted its role in stabilizing tRNA anticodon-codon interactions, its presence in mRNA, catalyzed by NAT10, significantly enhances transcript stability. This modification, primarily within coding sequences, reduces mRNA degradation and optimizes ribosomal decoding, establishing ac4C as a key regulator of post-transcriptional gene expression in human cells.^47^ Direct biophysical evidence for the duplex-stabilizing role of ac4C comes from thermodynamic analyses of RNAs containing the modification within physiological 5′-CCG-3′ motifs. These studies, conducted on human 18S rRNA and the D-arm of tRNA, demonstrated that ac4C significantly increases the melting temperatures of these transcripts.^48^ As demonstrated in mammalian models where the genetic ablation of the NAT10 adaptor THUMPD1 leads to loss of ac4C modification and consequent degradation of tRNA^Leu^(CAG) under thermal stress.^49^ Recent studies further reveal that ac4C also bolsters the structural integrity of DNA: RNA hybrids formed at genomic lesion sites. By acetylating the RNA component, NAT10 strengthens hybrid duplex stability, which in turn supports efficient DNA repair processes.^50^ This mechanism not only influences mRNA half-life but also contributes to genome maintenance under stress conditions.

In an HIV-1 infection model, NAT10-mediated modification of ac4C promotes the gene expression of viral RNAs by significantly enhancing their stability; inhibition of ac4C formation accelerates the degradation of viral transcripts, thereby impairing viral replication.^51^ Similarly, during the life cycle of the DNA virus KSHV, the virus co-opts the host NAT10 to mediate the ac4C modification of the viral long non-coding RNA: polyadenylated nuclear RNA (PAN RNA). This ac4C modification enhances PAN RNA stability and facilitates viral reactivation.^52^ In yeast, the absence of ac4C modification reduces tRNA stability by jeopardizing ac4C and Um modifications and Mg^++^ tertiary interactions, destabilizing the acceptor and T-stems, making tRNAs such as tRNA^Ser^ susceptible to rapid degradation by the 5′–3′ exonucleases Xrn1 and Rat1.^53^

### Primary microRNAs processing

A landmark study by Zhang et al. revealed that ac4C modification extends to primary microRNAs (pri-miRNAs), where it facilitates their maturation. NAT10, in complex with THUMPD1, catalyzes ac4C deposition on pri-miRNAs, particularly within stem-loop regions. This modification enhances the affinity of pri-miRNAs for the microprocessor component DGCR8, promoting efficient cleavage by DROSHA and subsequent pre-miRNA production.^24^

### R-loop resolution

R-loops are three-stranded nucleic acid structures composed of an RNA-DNA hybrid and a displaced single-stranded DNA strand, which form transiently during transcription. Although physiological R-loop formation is involved in key nuclear processes, its aberrant accumulation can induce transcription-replication conflicts, DNA double-strand breaks, and genomic instability. Timely resolution of R-loops is therefore essential for maintaining genome integrity. Beyond its previously described role in acetylating the RNA helicase DDX21 to stimulate R-loop unwinding,^12^ recent studies reveal that NAT10 also directly acetylates the RNA moiety within R-loops via ac4C modification. This epitranscriptomic mark is proposed to alter the structural conformation or stability of the RNA strand, facilitating R-loop resolution. Moreover, acetylation prevents the accumulation of unmodified R-loop-derived RNA fragments in endosomal compartments, thereby avoiding the activation of the single-stranded RNA sensor and subsequent pro-inflammatory signaling.^54^

Together, these studies establish ac4C as a multifunctional modulator of RNA fate (Figure 2C), thereby playing a pivotal role in post-transcriptional gene regulation. Its context-dependent effects underscore the sophistication of epitranscriptomic regulation.

## Research methodology of N4-acetylcytidine

### Chromatographic and mass spectrometry-based profiling

The detection and mapping of ac4C in RNA have posed significant technical challenges due to its low abundance and minimal impact on Watson-Crick base pairing. Early efforts relied on chromatographic and mass spectrometry-based techniques. High-performance liquid chromatography (HPLC) coupled with enzymatic digestion enabled the separation and identification of acetylated nucleosides, though with limited sensitivity and inability to resolve modifications at nucleotide resolution.^55^ Liquid chromatography-mass spectrometry offered improved quantification of ac4C from hydrolyzed RNA fragments, yet remained constrained by sample complexity and insufficient sensitivity for transcriptome-wide mapping.^14^^,^^56^ These foundational techniques are best suited for bulk quantification rather than high-resolution mapping, and their utility is limited by the requirement for large input RNA and extensive sample preprocessing.

### Antibody-based enrichment and acRIP-seq

A major advance arrived in 2017 with the development of antibody-based approaches. Sinclair et al. generated and validated a monoclonal anti-ac4C antibody through the conjugation of ac4C to carrier proteins and the generation of ac4C-containing RNA probes via *in vitro* transcription with synthetic ac4CTP. The study further established the specificity of this antibody by demonstrating its selective response to the chemical deacetylation of ac4C in cellular RNA using hydroxylamine treatment.^28^ The generation of ac4C-specific antibodies facilitated the immunoprecipitation-based enrichment of acetylated RNAs, leading to the establishment of acRIP-seq. This method enabled the first transcriptome-wide identification of thousands of ac4C sites in human mRNAs, revealing a bias toward coding sequences and 5′UTRs.^4^ However, acRIP-seq provides regional rather than single-base resolution and is susceptible to antibody-related artifacts and false positives. A critical limitation lies in the potential for antibody cross-reactivity or non-specific enrichment, especially for low-abundance modifications.^57^^,^^58^

### Chemical reduction strategies for base-resolution mapping

To achieve nucleotide-resolution mapping of ac4C, chemical reduction-based sequencing strategies were pioneered by Thomas et al. in 2018. The ac4C-seq technique employs sodium cyanoborohydride under acidic conditions to reduce ac4C to N-acetyl-3,4,5,6-tetrahydrocytidine, a derivative that preferentially base-pairs with adenosine during reverse transcription, thereby introducing C-to-T mismatches in the resulting cDNA. By computationally identifying sites with significant enrichment of C-to-T transitions in reduced samples compared to non-reduced or chemically deacetylated controls, this method enables the base-resolution identification of ac4C sites. However, inherent methodological challenges included pronounced variability in reduction efficiency between replicates, significant RNA degradation under standard reaction conditions, and an unexpectedly high background of thymine-associated misincorporation events. These factors can compromise quantification accuracy and sensitivity, particularly for low-stoichiometry sites.^59^^,^^60^ A related approach, RedaC:T-seq, utilizes sodium borohydride reduction under alkaline conditions. Although this reaction is also susceptible to inefficiencies and off-target deacetylation, the method achieves higher sequencing depth and significantly lower baseline error rates across all mismatch types. This enhanced signal-to-noise ratio allows for high-confidence mapping of ac4C sites, enabling reliable discrimination between acetylated and unmodified cytidines in both highly structured rRNA and polyadenylated mRNA.^46^^,^^61^ A significant advance was introduced with RetraC:T, which enhances C-to-T mismatch detection by incorporating the modified nucleotide 2-amino-dATP during reverse transcription. This analog preferentially base-pairs with tetrahydro-ac4C, driving near-stoichiometric mismatch rates at known ac4C sites and improving detection sensitivity even at low modification stoichiometries, without increasing off-target errors.^62^ More recently, tRNA reduction and misincorporation sequencing (TRMC-seq) has been developed to overcome limitations in detecting ac4C in low-abundance, highly modified tRNAs. By isolating tRNAs and optimizing reaction conditions, TRMC-seq achieves single-nucleotide resolution with improved sensitivity and specificity, enabling the discovery of previously unannotated ac4C sites in multiple tRNA isoacceptors.^36^ An overarching caveat for all reduction-based methods is their limited chemical specificity; other electron-deficient nucleobases can also undergo reduction, potentially generating false positives without carefully designed negative controls and comparative analysis in writer-deficient cells. Also, significant RNA degradation under chemical treatment may introduce biases in library preparation, especially for low-abundance transcripts.

### Emergence of direct RNA sequencing

Despite significant advancements in chemical-based methods for ac4C detection, concerns regarding specificity persist, as various modified nucleosides may undergo analogous reductive reactions, complicating accurate interpretation. In recent years, direct RNA sequencing via nanopore technology has emerged as a transformative approach for epitranscriptomic profiling. This technique enables real-time, amplification-free detection of RNA modifications by measuring alterations in ionic current as RNA molecules traverse the nanopore. Notably, modifications such as ac4C induce characteristic disruptions in current signals, which can be captured and decoded using advanced computational models.^63^

Building on this foundation, Wu et al. present a deep learning framework, termed modCnet, designed to concurrently identify ac4C and m^5^C modifications using nanopore direct RNA sequencing data.^64^ The model leverages both base-level features, such as mean current, signal deviation, dwell time, and base quality, and raw current signals corresponding to 5-mer contexts centered on cytidine residues. Through training on *in vitro* transcribed RNAs with defined modifications, modCnet achieved high accuracy in distinguishing ac4C from unmodified cytidine and other modifications. This approach circumvents many limitations of indirect detection methods, offering the potential for multiplexed modification mapping without chemical treatment or reverse transcription. Nevertheless, the accuracy of such computational tools remains contingent upon the diversity and quality of training data, highlighting the need for continued refinement and expansion of reference datasets to enhance predictive performance across varied biological contexts. Nevertheless, the accuracy of such computational tools remains contingent upon the diversity and quality of training data, highlighting the need for continued refinement and expansion of reference datasets to enhance predictive performance across varied biological contexts. A major current limitation is the difficulty in deconvoluting the signals from multiple, potentially adjacent modifications, and the performance can vary significantly with RNA sequence context and library preparation protocols.

### N4-acetylcytidine site prediction technology

In parallel with experimental techniques, a variety of computational prediction tools have been developed to facilitate genome-wide identification of ac4C sites, offering scalable and cost-effective alternatives to labor-intensive biochemical assays. Notable examples include PACES, which employs random forest classifiers trained on position-specific dinucleotide sequence profiles and k-nucleotide frequencies; XG-ac4C, which leverages eXtreme Gradient Boosting with features derived from electron-ion interaction pseudopotentials; DeepAc4C, a convolutional neural network model integrating both physicochemical properties and distributed nucleic acid representations; and the more recent Voting-ac4C, which incorporates a transformer-based RNAErnie pre-trained model alongside traditional sequence encodings in an ensemble learning framework.^65^^,^^66^^,^^67^^,^^68^^,^^69^^,^^70^ These tools have demonstrated progressive improvements in predictive performance across metrics such as AUC, accuracy, and Matthew’s correlation coefficient, highlighting the potential of machine learning and deep learning in capturing sequence determinants of ac4C modification. Nonetheless, the accuracy and generalizability of these models remain constrained by several inherent limitations. A primary challenge lies in the quality and completeness of the training datasets, which are often derived from acRIP-seq data with limited resolution and potential for false positives or negatives. The reliance on heuristically selected negative samples and sequence-based features may also overlook important structural, evolutionary, or transcriptional determinants of acetylation. Moreover, many models exhibit limited cross-species applicability and are vulnerable to context-specific biases. Therefore, while valuable for hypothesis generation and preliminary screening, computational predictions must be treated as probabilistic guides and require rigorous experimental validation, especially when investigating specific biological mechanisms or clinical correlations.

Together, these methodological advances have progressively enhanced our ability to detect, quantify, and map ac4C at increasingly higher resolution, providing critical tools for elucidating the functional roles of this epitranscriptomic mark in RNA biology and cancer. The choice of methodology should be guided by the specific research question, weighing factors such as required resolution, sample type and abundance, and need for absolute quantification.

## RNA N4-acetylcytidine modification in human cancers

The RNA modification ac4C functions not as a simple binary switch, but as a multifaceted regulator at the post-transcriptional crossroads. By modifying a broad spectrum of transcripts, it alters their fate and function, implicating it in the pathogenesis and progression of a diverse range of human diseases, including autoimmune and inflammatory disorders,^54^^,^^71^^,^^72^^,^^73^ infections,^74^ cardiac^75^^,^^76^^,^^77^ and neurological^78^^,^^79^ conditions, and bone diseases.^80^^,^^81^ This review will focus specifically on its roles and underlying mechanisms in human malignancies.

Cancer is characterized by a constellation of hallmarks, including sustained proliferative signaling, tissue invasion and metastasis, avoidance of immune destruction, evasion of apoptosis, and deregulating cellular energetics.^82^^,^^83^ Prior to 2018, a limited number of studies reported that NAT10 could reinforce malignant phenotypes in certain cancers, yet the underlying molecular mechanisms remained largely unexplored.^84^^,^^85^^,^^86^ The subsequent identification of the mRNA ac4C modification and concurrent advances in research methodologies have since catalyzed a deeper understanding of its functional implications in oncology. It is now evident that NAT10 is frequently upregulated in human cancers, and its role in mediating ac4C deposition is increasingly being decoded as a critical oncogenic mechanism.

### Cell proliferation

ac4C modification drives the rapid proliferation of tumor cells by directly or indirectly stabilizing mRNAs encoding oncogenes involved in cell cycle progression and growth signaling pathways.

NAT10-mediated ac4C modification of *MDM2* mRNA enhances its stability, leading to suppressed p53 expression in gastric cancer and facilitating G2/M phase progression and cell survival.^87^ ac4C modification within the CDS of *ERRFI1* mRNA by NAT10 increases its stability, resulting in the reactivation of the EGFR signaling pathway to drive proliferation.^43^

High-mobility group (HMG) proteins perform critical functions within the nucleus, such as maintaining chromatin architecture and facilitating DNA recombination. Extranuclearly, they orchestrate a myriad of biological processes, including cell differentiation, migration, metastasis, apoptosis, and inflammatory responses.^88^^,^^89^ NAT10 promotes cell cycle progression by stabilizing *HMGA1* mRNA via ac4C in prostate cancer.^90^ In HCC, ac4C modification in the CDS of *HMGB2* mRNA enhances the recruitment of the translation elongation factor eEF2, thereby boosting HMGB2 protein synthesis and driving tumor growth and metastasis.^91^

### Tissue invasion and metastasis

ac4C modification promotes tumor invasion and metastatic capability by upregulating factors associated with epithelial-mesenchymal transition (EMT) and extracellular matrix remodeling.

Cytoplasmic relocalization of NAT10 promotes EMT, characterized by E-cadherin downregulation and vimentin upregulation, enhancing HCC invasiveness. Conversely, NAT10 knockdown reduces DDIAS mRNA stability, attenuating cell proliferation, migration, and invasion via the PI3K/AKT pathway.^92^ In esophageal squamous cell carcinoma, NAT10-mediated ac4C modification stabilizes the long non-coding RNA CTC-490G23.2, which acts as a molecular scaffold, enhancing the interaction between CD44 pre-mRNA and the splicing factor PTBP1, leading to an oncogenic CD44 splice variant that promotes metastasis.^23^ Separately, NAT10 enhances the translation efficiency of the v-ATPase subunit ATP6V0E1 in an ac4C-dependent manner, leading to lysosomal acidification and promoting E-cadherin degradation, thereby facilitating invasion.^93^ NAT10 stabilizes the lncRNA SIMALR via ac4C modification, which binds to eukaryotic elongation factor 1α2 (eEF1A2), enhancing its GTPase activity and promoting the translation of ITGB4 and ITGA6, key players in cell adhesion and migration, thus fueling nasopharyngeal carcinoma progression.^22^

ac4C modification also influences organotropism and structural targeting during metastasis. In gastric cancer, ac4C modification of *KLF5* mRNA enhances its expression, leading to the transcriptional activation of integrin αV (ITGαV), which promotes gastric cancer cell attachment to hepatocytes and liver metastasis.^94^ In pancreatic ductal adenocarcinoma, ac4C modification within the coding sequence of ITGB5 mRNA increases its stability, activating the ITGB5–pFAK-pSrc pathway and facilitating perineural invasion.^95^ In clear-cell renal cell carcinoma, ac4C modified upregulated ANKZF1 interacts with YWHAE, promoting YAP1’s nuclear translocation, which activates the transcription of *VEGFC/D*, thereby driving tumor progression and lymphangiogenesis.^96^

### Metabolic reprogramming

By enhancing the stability and translation efficiency of mRNAs encoding metabolic enzymes and regulators, ac4C drives the alterations in glycolysis, fatty acid metabolism, and amino acid metabolism that fuel tumor growth and progression.

A key metabolic adaptation in cancer is the Warburg effect,^97^ wherein cancer cells preferentially utilize glycolysis for energy production even under aerobic conditions, resulting in lactate accumulation. ac4C modification directly influences the enzyme catalyzing its first step, hexokinase (HK).^44^^,^^98^ Furthermore, a NAT10/SEPT9/HIF-1α positive feedback loop is established in gastric cancer under hypoxia: HIF-1α upregulates NAT10, which acetylates *SEPT9* mRNA to enhance its stability, leading to sustained HIF-1 signaling and glycolytic addiction.^99^ In osteosarcoma, NAT10-mediated ac4C modification stabilizes *YTHDC1* mRNA. The elevated m6A reader YTHDC1 then enhances the translation of key glycolytic enzymes PFKM and LDHA in an m6A-dependent manner, illustrating a crosstalk between RNA modifications.^100^ In hepatoblastoma, Yes-associated protein 1 (YAP1) binds to the *NAT10* promoter to induce its transcription. NAT10 then stimulates ac4C modification within the 3′ UTR of glucose-6-phosphate dehydrogenase (G6PD) mRNA, enhancing its stability. This YAP1/NAT10/G6PD axis augments the pentose phosphate pathway (PPP), promoting hepatoblastoma proliferation and metastasis.^30^

Beyond glycolysis, ac4C plays a critical role in lipid metabolism. Dalhat et al. found that knockdown of NAT10 in cancer cells reveals a significant enrichment of downregulated genes in fatty acid metabolism pathways. NAT10 stabilizes mRNAs of crucial lipid metabolism genes (e.g., ELOVL6, ACSL1/3/4, ACADSB, and ACAT1) via ac4C, maintaining lipid, triglyceride, and cholesterol levels. Conversely, NAT10 depletion disrupts this process, leading to an accumulation of polyunsaturated fatty acids (PUFAs) and oxidized phospholipids, potentially inducing ferroptosis.^101^ In ovarian cancer, m6A-driven NAT10 upregulation increases *ACOT7* mRNA ac4C modification, mediating tetradecenoic acid (C14:1) production and inhibiting ferroptosis.^102^ Similarly, in esophageal squamous cell carcinoma, exosomal NAT10 stabilizes the expression of fatty acid synthase (FASN) and promotes macrophage M2 polarization by facilitating lipid metabolism.^103^

ac4C also influences amino acid metabolism. In osteosarcoma, ac4C-mediated upregulation of ATF4, a master regulator of the integrated stress response, induces the transcription of asparagine synthetase (ASNS). This enhances asparagine biosynthesis, thereby supporting anabolic demands and driving tumor progression.^104^

### Avoiding immune destruction

ac4C modification enables tumor cells to evade immune surveillance through multiple mechanisms, dysregulating the tumor microenvironment and the immune checkpoint pathways.

Metabolic reprogramming toward glycolysis results in lactate accumulation, fostering an immuneosuppressive tumor microenvironment (TME). In triple-negative breast cancer (TNBC), NAT10 stabilizes *JunB* mRNA via ac4C, leading to upregulated LDHA expression, enhanced glycolysis, and lactate production, which creates an acidic, immunosuppressive TME.^105^ Similarly, in cervical cancer, NAT10-mediated ac4C modification stabilizes FOXP1 mRNA, upregulating GLUT4 and KHK to reprogram metabolism toward glycolysis and lactate secretion, fostering an immunosuppressive microenvironment via Treg activation.^106^

ac4C modification also shapes the immune landscape by modulating cytokine networks. The RNPS1-NAT10 axis enhances the translation of pro-tumorigenic cytokines such as IL-6 and IL-8 via ac4C modification. These cytokines help establish an immunosuppressive TME by recruiting immunosuppressive cells and dampening cytotoxic T cell activity.^36^ The NAT10/ac4C/DDX5 axis upregulates HMGB1 and suppresses CD4^+^ and CD8^+^ T cell activity via cytokines CXCL9, CXCL10, and CXCL16, further impairing PD-1 therapy sensitivity of nasopharyngeal carcinoma.^107^ In gastric cancer, *CXCL2* mRNA is stabilized by ac4C modification, and then secreted CXCL2 promotes the infiltration and polarization of M2-like macrophages.^94^

Furthermore, ac4C influences key signaling pathways that modulate the immune response. NAT10-dependent ac4C stabilizes the lncRNA XIST, which recruits hnRNPK to promote YAP1 nuclear translocation and VEGFA transcription, leading to abnormal, hypoxic vasculature that restricts cytotoxic lymphocyte infiltration while enriching Tregs.^108^ NAT10 stabilizes LAMB3 mRNA via ac4C, activating the FAK/ERK pathway, upregulating PD-L1 expression on tumor cells, enhancing PD-1/PD-L1 interaction, and CD8^+^ T cell inhibition.^109^

### Therapy resistance

A key mechanism involves the potentiation of DNA repair pathways. In HCC, NAT10-mediated ac4C stabilizes DNA:RNA hybrids at double-strand break sites, promoting homologous recombination repair and conferring resistance to PARP inhibitors.^50^ In breast cancer, NAT10 upregulates *RAD51* mRNA stability via ac4C, leading to increased homologous recombination efficiency and resistance to olaparib.^110^ In bladder cancer, cisplatin induces the NAT10-mediated ac4C modification of *AHNAK* mRNA, enhancing its stability and promoting DNA damage repair, contributing to cisplatin resistance.^111^

Resistance to small molecule targeted therapies and radiotherapy can also be facilitated by ac4C. ac4C modification stabilizes *HSP90AA1* mRNA, enhancing cell survival under endoplasmic reticulum stress and conferring HCC resistance to lenvatinib-induced apoptosis.^112^ Furthermore, stabilization of *SLC7A11* mRNA, a key component of the glutathione synthesis pathway, inhibits sorafenib-induced ferroptosis, promoting nasopharyngeal carcinoma cells' survival.^113^ In non-small cell lung cancer, KPNB1 expression is upregulated and promotes radiotherapy resistance by facilitating PD-L1 nuclear translocation.^114^

**Figure 4:**
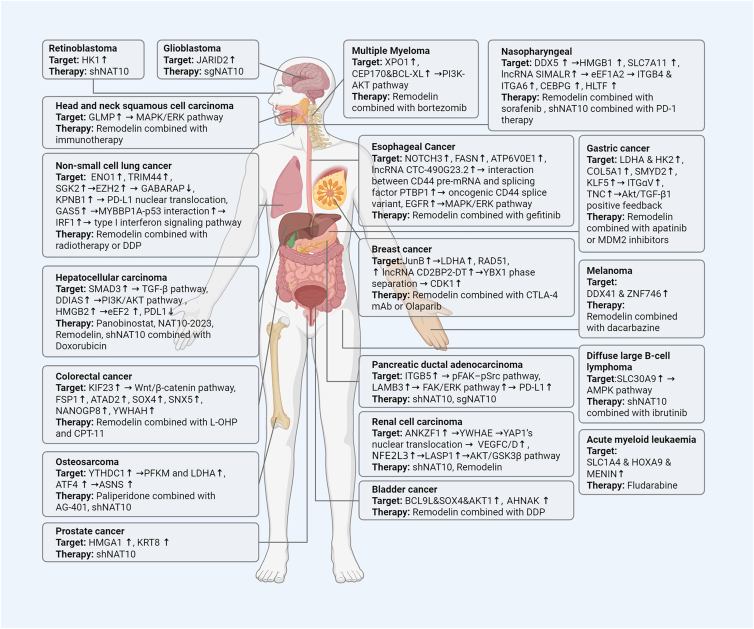
Organ-specific landscape of ac4C-modified transcripts and their therapeutic implications in human cancers The schematic human model summarizes the key ac4C-regulated oncogenic targets across different cancer types, their roles in tumor pathogenesis within specific organs, and the corresponding therapeutic strategies. In the Target column: upward (↑) and downward (↓) arrows denote the upregulation or downregulation, respectively, of specific mRNA/protein expression. Rightward arrows (→) indicate the activation of downstream signaling pathways or molecular consequences. The Therapy column lists therapeutic strategies, including pharmacological inhibitors and rational combinations that target the NAT10-ac4C axis and have shown efficacy in preclinical models for the respective cancer types.

The roles of NAT10-mediated RNA ac4C modification across different cancers are summarized in Table 1 and Figure 4.

**Table 1:** Summary of ac4C-mediated mechanisms in human cancers

Cancer type	Target	Mechanism	Reference
Acute myeloid leukemia	SLC1A4, HOXA9, MENIN	Increases cellular uptake of serine and facilitates the transcription of serine synthesis pathway genes for leukemogenesis and stemness	Zhang et al.^115^
Bladder cancer	BCL9L, SOX4, AKT1	Promotes tumor proliferation and invasion	Wang et al.^116^
Bladder cancer	AHNAK	Promotes DNA damage repair, contributing to cisplatin resistance	Xie et al.^111^
Breast cancer	lncRNA CD2BP2-DT	CD2BP2-DT-driven YBX1 phase separation enhances CDK1 mRNA stability and thus promotes breast cancer cell proliferation	Wang et al.^117^
Clear cell renal cell carcinoma	ANKZF1	Upregulated ANKZF1 interacts with YWHAE and promotes YAP1’s nuclear translocation, activating the transcription of VEGFC/D, thereby driving tumor progression and lymphangiogenesis	Miao et al.^96^
Clear cell renal cell carcinoma	NFE2L3	The transcription factor NFE2L3 binds to the promoter region of LASP1 to regulate its expression and activates the LASP1-AKT/GSK3β pathway to promote tumor proliferation and metastasis	Sun et al.^118^
Cervical cancer	YAP	Promotes tumor proliferation and metastasis	Zhang et al.^41^
Cervical cancer	SLC7A5	Promotes tumor proliferation and metastasis	Liang et al.^119^
Colorectal cancer	FSP1	Inhibits ferroptosis triggered by lipid peroxidation, promotes tumor proliferation and metastasis	Zheng et al.^120^
Colorectal cancer	KIF23	Up-regulated KIF23 activates the Wnt/β-catenin pathway and promotes tumor proliferation, migration, and invasion	Jin et al.^121^
Colorectal cancer	ATAD2, SOX4, SNX5	Promotes tumor metastasis and angiogenesis	Song et al.^34^
Colorectal cancer	NANOGP8	Inhibits tumor chemosensitization and promotes stemness	Gao et al.^122^
Colorectal cancer	YWHAH	Associates with immune checkpoint proteins, induces CD8^+^ T cell exhaustion, characterized by decreased proliferation and increased exhaustion markers	Li et al.^123^
Diffuse large B-cell lymphoma	SLC30A9	Promotes tumor proliferation by regulating the activation of the AMP-activated protein kinase (AMPK) pathway	Ding et al.^124^
Esophageal Cancer	EGFR	Activates MAPK/ERK signaling pathway, promotes cell proliferation and gefitinib resistance	Wei et al.^125^
Esophageal Cancer	NOTCH3	Enhances fibronectin expression and promotes tumor metastasis	Liao et al.^35^
Gastric cancer	LDHA, HK2	Promotes tumor proliferation and glycolysis	Wang et al.^44^
Gastric cancer	COL5A1	Promotes tumor EMT and metastasis	Zhang et al.^126^
Gastric cancer	TNC	Activates TNC/Akt/TGF-β1 positive feedback, promotes tumor progression	Chen et al.^127^
Gastric cancer	SMYD2	Promotes tumor migration and invasion	Liu et al.^128^
Glioblastoma	JARID2	Promotes tumor stemness	Inoki et al.^129^
Hepatocellular carcinoma	PDL1	Promotes PDL1 degradation, prevents MDSCs differentiation, and enhances CTL infiltration	Xu et al.^130^
Hepatocellular carcinoma	SMAD3	Activates the TGF-β signalling pathway to drive tumor progression	Zhang et al.^131^
Head and neck squamous cell carcinoma	GLMP	Activates the MAPK/ERK pathway to promote lymph node metastasis and tumor microenvironment remodeling	Liu et al.^132^
Melanoma	DDX41, ZNF746	Promotes chemoresistance to dacarbazine	Wang et al.^133^
Multiple Myeloma	XPO1	Promotes bortezomib resistance	Xu et al.^134^
Multiple Myeloma	CEP170, BCL-XL	Activates PI3K-AKT pathway, promotes tumor proliferation	Wei et al.^135^
Nasopharyngeal carcinoma	lncRNA SIMALR	Upregulated SIMALR binds to eukaryotic elongation factor 1α2 (eEF1A2), enhancing its GTPase activity and the translation of ITGB4 and ITGA6, promoting tumor cell adhesion and migration	Gong et al.^22^
Nasopharyngeal carcinoma	FOXD2	Upregulated expression of FODX2 acted as a transcriptional activator of NAT10, promoting tumor cell proliferation and invasion	Liu et al.^136^
Non-small cell lung cancer	KPNB1	Promotes radiotherapy resistance by facilitating PD-L1 nuclear translocation	Zhu et al.^114^
Non-small cell lung cancer	ENO1	Promotes tumor glycolysis, inhibits apoptosis	Yuan et al.^137^
Non-small cell lung cancer	TRIM44	Activates PI3K/AKT pathway, promotes DDP resistance	Sun et al.^138^
Non-small cell lung cancer	SGK2	Stabilizes EZH2, inhibits GABARAP transcription, and hinders autophagy	Xiao et al.^139^
Non-small cell lung cancer	GAS5	Promotes MYBBP1A-p53 interaction, upregulates IRF1 expression, activates type I interferon signaling pathway, induces chemokine secretion, and attracts macrophages and T cells to infiltrate the tumor microenvironment	Wang et al.^140^
Ovarian cancer	CAPRIN1	Promotes tumor migration, invasion, and stemness	Song and Cheng^141^
Pancreatic ductal adenocarcinoma	ITGB5, AXL	Activates the ITGB5–pFAK-pSrc pathway, promotes tumor proliferation, metastasis, and perineural invasion	Huang et al.^95^
Prostate cancer	HMGA1, KRT8	Promotes cell cycle progression to improve tumor proliferation	Li et al.^90^
Retinoblastoma	HK1	Promotes tumor glycolysis	Xu et al.^98^

## Potential clinical value and future prospects

### Therapeutic targeting of the N-acetyltransferase 10-N4-acetylcytidine axis

The growing understanding of RNA modifications has consistently demonstrated a path from mechanistic discovery at the bench to clinical application at the bedside. In the case of the NAT10–ac4C axis, burgeoning evidence of its critical role in human pathologies, including cancer, underscores its significant potential as a target for therapeutic intervention. However, the clinical translation of this knowledge extends beyond inhibitor development and encompasses diagnostic, prognostic, and combinatorial strategies.

The most direct therapeutic approach is the pharmacological inhibition of NAT10. The small molecule Remodelin was initially identified in the context of Hutchinson-Gilford progeria syndrome for its ability to restore nuclear architecture in laminopathic cells by perturbing microtubule anchoring at the centrosome, thereby alleviating mechanical stress on the nuclear envelope.^7^ Since its repurposing in 2018 to reverse doxorubicin resistance by suppressing epithelial-mesenchymal transition in breast cancer,^142^ Remodelin has served as a foundational proof-of-concept tool in preclinical studies. It has demonstrated efficacy across diverse malignancies, including pancreatic ductal adenocarcinoma, hepatoblastoma, gastric cancer, and esophageal squamous cell carcinoma, where it suppresses tumor growth and metastasis, and sensitizes cancer cells to chemotherapy, radiotherapy, and targeted agents in cellular and animal models.^30^^,^^94^^,^^109^^,^^112^^,^^125^ Given the prominent role of the NAT10–ac4C axis in fostering broad-spectrum therapy resistance, its targeting is unlikely to succeed as a monotherapy but rather could powerfully resensitize tumors to established agents. This rationale has spurred the evaluation of rational combinatorial therapies in preclinical settings. Notable examples include the combination of NAT10 inhibition with PARP inhibitors,^50^ immunotherapy,^105^^,^^106^^,^^110^ chemotherapeutic agents^10^^,^^111^^,^^122^ and tyrosine kinase inhibitors.^99^^,^^113^ Such strategies are critical for overcoming entrenched resistance mechanisms. It is crucial to emphasize, however, that these compelling findings remain confined to preclinical models, which cannot fully recapitulate the complexity of the human tumor microenvironment and systemic physiology. Consequently, the translational promise of targeting the NAT10–ac4C axis must now be validated in future clinical trials. Figure 4 summarizes the current landscape of pharmacological agents targeting NAT10.

However, the designation of Remodelin as a specific NAT10 inhibitor has been contested. Shrimp et al. demonstrated that Remodelin should not be considered a selective chemical probe for NAT10, citing a lack of robust biophysical evidence for its direct interaction with the NAT10 acetyl-CoA binding pocket and its failure to suppress NAT10’s core biological function: the acetylation of RNA to form ac4C.^143^ However, a recent cryo-electron microscopy study resolved the structure of NAT10 in complex with Remodelin at 2.9 Å resolution, revealing direct binding of Remodelin adjacent to the acetyl-CoA binding cavity and detailing specific interactions with residues such as H539, L606, S714, and S747.^50^ These unresolved questions concerning its precise mechanism, direct target engagement, and functional selectivity underscore the necessity for developing more potent and specific inhibitors. In the course of investigating NAT10’s mechanisms, other pharmacological agents have emerged as potential modifiers of its function. The anti-leukemic agent Fludarabine has been repurposed as a novel small-molecule inhibitor of NAT10.^115^ Similarly, the histone deacetylase inhibitor Panobinostat has been validated as a potent suppressor of NAT10-mediated ac4C modification, significantly attenuating the growth and metastasis of HCC *in vivo*.^91^ Intriguingly, drug repurposing efforts have also identified the atypical antipsychotic Paliperidone as a binder of the NAT10 protein. In osteosarcoma, Paliperidone reduces global ac4C modification levels on mRNA, thereby suppressing tumorigenesis by diminishing the stability and expression of key oncogenic transcripts.^104^ Recently, the novel inhibitor NAT10-2023 was identified through virtual screening as a highly specific binder of NAT10, demonstrating the potent suppression of ac4C modification and disruption of NAT10–RNA interactions. In preclinical HCC models, it effectively impaired tumor growth and metastasis by decreasing oncogenic mRNA stability.^131^ Furthermore, virtual screening of FDA-approved drug libraries has suggested the repurposing potential of compounds such as Fosaprepitant, Leucovorin, and Dantrolene as putative NAT10 inhibitors.^144^ Beyond small molecules, foundational studies frequently utilize genetic tools such as RNA interference (shRNA) and CRISPR/Cas9 to achieve targeted NAT10 knockdown,^92^^,^^95^^,^^125^ providing powerful means for mechanistic dissection and hinting at future gene therapy applications.

A major hurdle is designing NAT10 inhibitors that do not disrupt its essential physiological functions in rRNA and tRNA modification, which could lead to on-target toxicity. Tissue-specific or subcellular-localization-specific delivery systems may be required. Looking forward, research efforts should capitalize on advanced strategies, including structure-based drug design informed by high-resolution NAT10 structures and AI-driven virtual screening, to identify novel compounds that unequivocally target the ac4C catalytic domain. Coupled with nanotechnology-based targeted delivery systems, the therapeutic objective would be to disrupt the oncogenic RNA regulon governed by NAT10, thereby reversing the ac4C-mediated stabilization of key drivers underpinning tumor progression and therapy resistance.

### Detection of N4-acetylcytidine modification as a diagnostic biomarker

Dysregulation of ac4C modification has been documented across multiple cancer types, highlighting its potential as a novel class of pan-cancer biomarkers. The assessment of ac4C levels or specific ac4C-modified RNAs in patient-derived samples holds considerable promise for non-invasive early detection, molecular subtyping, and prognostic stratification, enabling the identification of individuals with aggressive disease phenotypes, elevated metastatic potential, or heightened risk of relapse. Moreover, dynamic monitoring of ac4C signatures could inform therapeutic responses to PARP inhibitors or immunotherapy, paving the way for personalized treatment strategies. Liquid biopsy platforms detecting ac4C-modified transcripts in circulating tumor cells or exosomes from blood and other biofluids (e.g., ascites and cerebrospinal fluid) represent particularly promising avenues for clinical translation.

However, the clinical application of ac4C as a biomarker is constrained by current technological limitations in detecting this modification with high sensitivity, specificity, throughput, and single-nucleotide resolution from often scarce clinical specimens. For example, while molecularly imprinted solid-phase extraction has been used to selectively enrich endogenous pyrimidine nucleosides from urine, thereby enhancing HPLC detection sensitivity, the method’s alkaline extraction conditions lead to ac4C hydrolysis, precluding its accurate quantification.^145^ Future methodological advances are critically needed, such as improved third-generation sequencing platforms (e.g., refined nanopore sequencing) and antibody-free chemical detection techniques to enable base-resolution mapping of ac4C sites.^146^^,^^147^^,^^148^ In parallel, the growing accessibility of single-cell transcriptomics calls for the development of single-cell ac4C sequencing methods to dissect cell-to-cell heterogeneity of this modification within tumors and complex tissues. These technological strides will be essential to transition ac4C biomarker research from bulk analyses to the study of rare cell populations and limited clinical samples, ultimately integrating ac4C assessment into routine diagnostic pathology.

Complementing these experimental advances, computational tools are poised to play an increasingly vital role. As highlighted throughout this review, machine learning and artificial intelligence (AI)-based frameworks will be indispensable for predicting novel ac4C sites, integrating multi-omics data including epitranscriptomic, transcriptomic, and proteomic layers, and identifying patient subgroups based on ac4C modification patterns. Continued refinement of feature representation, adoption of explainable AI models, and validation through multi-omic datasets will be essential to enhance the biological relevance and predictive power of computational approaches in epitranscriptome annotation and clinical translation.

### Expanding the artificial intelligence regulatory network

The relatively recent discovery of ac4C RNA modification means fundamental aspects of its regulatory machinery remain incompletely understood. A profound gap exists in identifying the putative “reader” and “eraser” proteins responsible for recognizing and reversing this modification, respectively. The discovery of such regulators is paramount, as readers would determine the functional consequences of ac4C, while erasers would provide dynamic control and represent alternative therapeutic targets, akin to FTO^149^ and ALKBH5^150^ inhibitors in m6A biology. Furthermore, the full complement of essential and non-essential cofactors required for ac4C deposition remains undefined,^17^ yet represents a promising, albeit unexplored, avenue for therapeutic targeting. Elucidating these components will unveil a more complex and dynamic regulatory network, opening entirely new dimensions for therapeutic manipulation.

The biological role of the NAT10-ac4C axis exhibits remarkable context dependency. While it predominantly functions as an oncogene across diverse cancers, emerging evidence suggests potential tumor-suppressive roles in specific settings. In the immunotherapy of HCC, ac4C modification promotes the infiltration of M1-type macrophages while reducing the infiltration of myeloid-derived suppressor cells, thereby enhancing cytotoxic T lymphocyte-mediated tumor cell killing capacity.^130^ This functional duality may be dictated by the cellular and microenvironmental context, such as tumor type, genetic background, or the specific immune milieu. Further studies delineating the molecular and contextual determinants of these opposing outcomes will be crucial for understanding the full therapeutic potential of targeting the ac4C pathway. Beyond oncology, NAT10 mediates critical processes in non-malignant conditions, including inflammatory responses and tissue development. Understanding the determinants of these pleiotropic functions dictated by cell type, tissue microenvironment, or genetic background will be crucial for rational patient stratification and minimizing off-target effects in future therapeutic applications.

The functional interplay between ac4C and other RNA modifications, as well as classical epigenetic marks, represents a frontier. Preliminary evidence, such as the ac4C-dependent regulation of the m6A readers, hints at a sophisticated, multi-layered regulatory code.^100^ Deciphering the principles governing this epitranscriptomic cross-talk will be essential for a holistic understanding of its integrated control over gene expression in both physiological and pathological states.

## Conclusion

The ac4C modification, occurring across tRNA, rRNA, and mRNA, and catalyzed exclusively by the writer protein NAT10, constitutes a crucial and previously underappreciated layer of post-transcriptional regulation. While challenges remain, particularly in drug development and detection technology, the axis presents a multifaceted clinical opportunity. Future research focused on elucidating its full regulatory circuitry, developing sensitive diagnostic tools, and advancing targeted inhibitors holds the promise of integrating ac4C modification into the future of precision oncology, ultimately improving patient outcomes.

## Acknowledgments

This work was supported by grants from the 10.13039/501100001809National Natural Science Foundation of China (No. 82273132), Key Research and Development Program of Zhejiang (No. 2025C02071).

## Author contributions

Y.S.: writing – original draft, conceptualization, and investigation. J.S.: writing – original draft, conceptualization, and investigation. H.C.: writing – original draft and investigation. K.Y.: writing – original draft. J.L.: writing – review and editing, conceptualization, and supervision. B.L.: writing – review and editing, supervision, and funding acquisition. All authors have read and approved the final article.

## Declaration of interests

The authors declare no competing interests.
